# Valorization of homogeneous linear catechyl lignin: opportunities and challenges

**DOI:** 10.1039/d3ra01546g

**Published:** 2023-04-24

**Authors:** Yibing Li, Xianzhi Meng, Rongqian Meng, Ting Cai, Yunqiao Pu, Zhi-Min Zhao, Arthur J. Ragauskas

**Affiliations:** a School of Ecology and Environment, Inner Mongolia Key Laboratory of Environmental Pollution Control & Wastes Reuse, Inner Mongolia University Hohhot 010021 China zmzhao@imu.edu.cn; b Department of Chemical & Biomolecular Engineering, University of Tennessee Knoxville TN 37996 USA aragausk@utk.edu; c Inner Mongolia Autonomous Region Agriculture and Animal Husbandry Technology Extension Center Hohhot 010010 China; d Center for Bioenergy Innovation (CBI), Joint Institute of Biological Science, Biosciences Division, Oak Ridge National Laboratory Oak Ridge TN 37831 USA; e Center for Renewable Carbon, Department of Forestry, Wildlife, and Fisheries, University of Tennessee Institute of Agriculture Knoxville TN 37996 USA

## Abstract

Lignin is the dominant aromatic renewable polymer on earth. Generally, its complex and heterogeneous structure hinders its high-value utilization. Catechyl lignin (C-lignin), a novel lignin discovered in the seed coats of vanilla and several members of Cactaceae, has received increasing attention due to its unique homogeneous linear structure. Obtaining substantial amounts of C-lignin either by gene regulation or effective isolation is essential to advance C-lignin's valorization. Through a fundamental understanding of the biosynthesis process, genetic engineering to promote the accumulation of C-lignin in certain plants was developed to facilitate C-lignin valorization. Various isolation methods were also developed to isolate C-lignin, among which deep eutectic solvents (DESs) treatment is one of the most promising approaches to fractionate C-lignin from biomass materials. Since C-lignin is composed of homogeneous catechyl units, depolymerization to produce catechol monomers demonstrates a promising way for value-added utilization of C-lignin. Reductive catalytic fractionation (RCF) represents another emerging technology for effective depolymerizing C-lignin, leading to a narrow distribution of lignin-derived aromatic products (*e.g.*, propyl and propenyl catechol). Meanwhile, the linear molecular structure predisposes C-lignin as a potential promising feedstock for preparing carbon fiber materials. In this review, the biosynthesis of this unique C-lignin in plants is summarized. C-lignin isolation from plants and various depolymerization approaches to obtaining aromatic products are overviewed with highlights on RCF process. Exploring new application areas based on C-lignin's unique homogeneous linear structure is also discussed with its potential for high-value utilization in the future.

## Abstract 2

The discovery and valorization of the unique homogeneous linear catechyl lignin (C-lignin) are systematically summarized.

## Graphical abstract

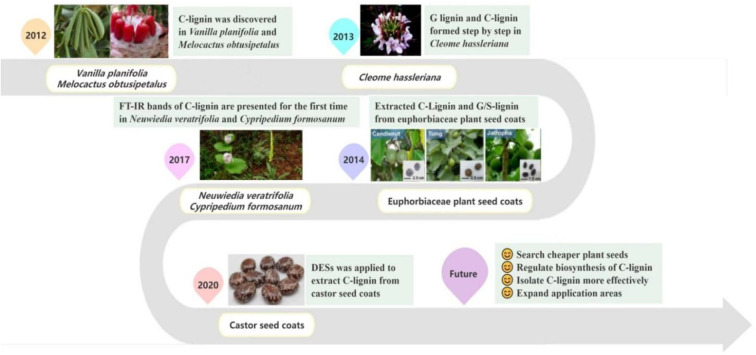

## Introduction

Lignin is a complex polymer that widely exists in various types of plants in nature. Its abundant functional groups (*e.g.*, hydroxyl and carboxyl groups) and aromatic nature offer great potential for high-value utilization.^1^ Generally, plants transform phenylalanine through aromatic hydroxylation and *O*-methylation to produce lignin monomers with different degrees of methoxylation, which are classified into syringyl units (S), guaiacyl units (G), and *p*-hydroxyphenyl units (H). These lignin monomers are conjugated through various ether or carbon–carbon bonds to form lignin macromolecules. In recent years, the application areas of lignin have been expanded to adsorbents,^2^ fertilizers,^3^ epoxy resin curing agents,^4^ lipids,^5^ polyhydroxyalkanoates (PHA),^6^ and polyurethanes,^7^*etc.* Despite these developments, it is estimated that only 2% of the total industrial lignin stream is currently used for preparing derivative products, while the major part is subjected to combustion or abandoned in landfills. The low efficient utilization of lignin mainly owes to its complex and inhomogeneous molecular structure.^8,9^

**Fig. 1:**
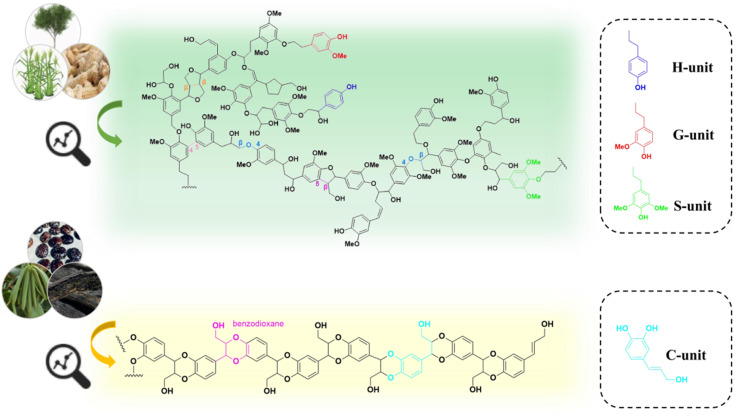
Proposed structure of H/G/S lignin *versus* C-lignin revealed by NMR analysis.^15^

**Fig. 2:**
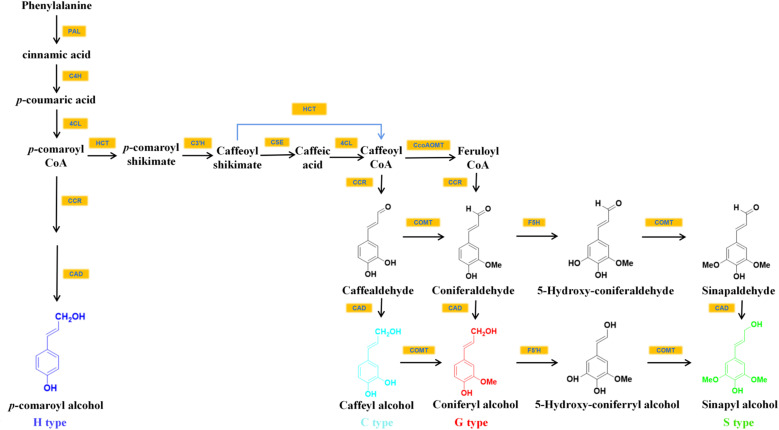
The inferred biosynthetic pathways of C-lignin and G/S/H lignin.^20–22^

It has been reported that various phenolic compounds are possible substrates that can be transformed into lignin units that are different from those typical lignin subunits (*e.g.*, S, G, and H). Ralph and coworkers elucidated the presence of a COMT (caffeic acid *O*-methyltransferase) defect poplar by NMR analysis of the lignin.^10^ In 2012, Chen and coworkers detected the presence of a novel lignin, catechyl lignin (C-lignin), in the seed coats of *Vanilla planifolia* and several members of the Cactaceae (*e.g.*, *Melocactus obtusipetalus*).^11^ C-lignin is formed by the free coupling of oxidized radicals, resulting in a linear polymer composed of caffeyl alcohol (Fig. 1 and 2).^11–14^ During plant growth, the lack of *O*-methyltransferase (OMT) activity leads to the selective formation of caffeyl alcohol monomers. The C_5_–OH in caffeyl alcohol facilitates monomer coupling with β-*O*-4 radicals to form intramolecular closed loops, resulting in homopolymers of C-lignin without condensation units (almost exclusively linked by benzodioxane bonds).^15–17^ Compared to the typical G/S lignin, C-lignin has a lower molecular weight, probably due to the weak polymerization ability of caffeyl alcohols compared to G/S lignin units.^11,13^ According to the analysis of the 3D structure by all-atom molecular dynamics simulation, C-lignin was found to be more dense and rigid.^18^ In addition, C-lignin shows good acid stability due to the stable benzodioxane structure.^17^

## Biosynthesis of C-lignin

### C-lignin biosynthesis in nature

Among all plants capable of accumulating C-lignin in the seed coats, the seed coats of Vanilla and Cactaceae contained only C-lignin, while those of Euphorbiaceae, Cleomaceae, the orchid families *N. veratrifolia* and *C. formosanum* contained C-lignin and conventional G/S-type lignin.^14,19^ In the seed coat of these crops where C-lignin and G/S-type lignin coexist, the synthesis of C-lignin and the synthesis of conventional lignin units are temporally independent of each other. For example, in *Cleome hassleriana*, G-lignin is synthesized in the seed coat shortly after pollination and stopped around 14 days after pollination. Afterward, the formation of C-lignin begins.^13,19^

The biosynthesis of monolignols begins with phenylalanine, which is converted to *p*-coumaroyl-CoA *via* the general phenylpropane pathway with the catalysis of l-phenylalanine ammonia-lyase (PAL), cinnamate 4-hydroxylase (C4H), and 4-coumarate-CoA ligase (4CL) (Fig. 2). *p*-Coumaroyl-CoA was then converted to caffeoyl shikimate with the help of catalysis by shikimate/quinate hydroxycinnamoyl transferase (HCT) and coumaroyl shikimate 3′-hydroxylase (C3′H). This biosynthetic pathway presumably leads to the introduction of the 3-hydroxyl group of the caffeoyl portion of C-lignin.^20,21^ Caffeoyl-CoA, one of the commonly used substrates for G/S lignin synthesis in angiosperms,^21^ is a direct precursor for the synthesis of caffealdehyde. Caffeoyl-CoA can be formed from caffeoyl shikimate either by the reverse HCT reaction or by the combined action of caffeoyl shikimate esterase (CSE) and 4CL.^20^ The loss of activity of CCoAOMT (caffeoyl-CoA 3-*O*-methyltransferase) and COMT enzymes leads to the selective formation of the C-lignin precursor substance caffeyl alcohol in plants. For example, vanilla seed coats contain C-lignin in high purity because they almost do not contain CCoAOMT transcript that could convert caffeoyl to feruloyl by methylation of hydroxyl groups to methoxy.^13,19^ Wagner and colleagues also reported that the silencing of the CCoAOMT gene resulted in the accumulation of C-lignin in *Pinus radiata*.^21^

It was found that in *Pinus radiata*, cinnamoyl CoA reductase (CCR) and cinnamyl alcohol dehydrogenase (CAD) were able to convert caffeoyl-CoA to caffealdehyde and caffeyl alcohol.^20,21^ ChCAD5, a form of cinnamyl alcohol dehydrogenase with a preference for caffeyl alcohol generation, could facilitate C-lignin synthesis. ChCAD5's substrate preference for caffealdehyde is influenced by His58 (histidine residue at position 58 of ChCAD5) and Lys60 residues (lysine residue at position 60 of ChCAD5). His58 and Lys60 increased the enzyme's affinity with caffealdehyde by interacting with the 3-OH group on caffealdehyde.^20^ It was demonstrated that the effect of C-lignin synthesis could be improved by increasing the substrate preference for caffealdehyde by increasing the level of ChCAD5's transcription.^20,23^ ChCAD4, another CAD gene associated with C-lignin biosynthesis, prefers coniferaldehyde over caffealdehyde substrates, which presents decreased expression during C-lignin accumulation.^20^

In addition, some specific laccase enzymes are associated with C-lignin synthesis. A seed coat-specific laccase from *Cleome hassleriana* (ChLAC8, which oxidizes caffeyl alcohol) exhibits an expression profile similar to the accumulation pattern of C-lignin during seed maturation. The appearance of C-lignin, along with the expression of ChLAC8 in COMT mutants of *Medicago truncatula* and *Arabidopsis*, suggests that this enzyme promotes the polymerization of caffeyl alcohol in plants.^23^ The substrate specificity of these polymerases associated with the polymerization of lignin monomers still needs to be revealed. Their effects on lignin composition remain unclear. Searching for more specific peroxidase enzymes for C-lignin polymerization represents a promising approach to help the accumulation of C-lignin in the target plants.

### Regulation of C-lignin synthesis

Currently, the primary sources of C-lignin are vanilla seeds ($350–500 per kg) and castor seed coats ($0.5–1 per kg).^24^ In addition, C-lignin exists in the seed coats of *N. veratrifolia* and *C. formosanum* of the orchid families.^14^ Vanilla seeds are rich in C-lignin, however, they are expensive. Besides C-lignin, G/S-type lignin also exists in castor seed coats. The size of Orchid seeds is small, resulting in difficulties in the processing to extract C-lignin. Moreover, impurities such as lipids, polysaccharides, and waxes exist in plant seed coats, which require an additional operation to avoid contamination during the subsequent valorization of C-lignin. Improving C-lignin yield and reducing economic cost are crucial in expanding the prospect of C-lignin utilization. Genetic engineering to regulate lignin unit synthesis represents a promising way to address this issue.

It is feasible to modulate the lignin monomer synthesis process to obtain the desired C-lignin. For example, inhibition of CCoAOMT gene expression in *Pinus radiata* resulted in C-lignin accumulation.^21^ However, not all plants can realize caffeyl alcohol binding and benzodioxin bond production by inhibiting CCoAOMT and COMT, such as *Arabidopsis*, *Medicago sativa*, poplar (*Populus tremula* × *Populus alba*), and *Nicotiana tabacum*.^25–29^ Moreover, some mutants even show impaired growth or decreased total lignin content.^22,27,30^ The lack of precursors is one reason why plants cannot accumulate C-lignin by simply inhibiting the relevant *O*-methyltransferase genes. Promotion of the genes that can introduce caffeyl alcohol precursors might solve the problems of reduced lignin content and plant growth defects caused by inhibition of COMT or CcoAOMT genes.^20,31^ Several factors may interfere with the binding of caffeyl alcohol in lignin polymers. For example, caffeyl alcohol is highly reactive and may be oxidized by polyphenol oxidases and catechol dioxygenases. In addition, caffeoyl CoA has the potential to be introduced into pathways like flavonoid biosynthesis, which may affect the production of caffeyl alcohol.^21^ Some plants with suppressed CCoAOMT and COMT gene expression still do not accumulate C-lignin, suggesting that the blockage of the *O*-methylation process may be somewhat detrimental to plant growth if it is not balanced by a reduction in methyl supply. Understanding these limitations is essential for the rational design of plants as platforms for C-lignin production.^20^ In fact, the products of the shikimate and phenylpropane pathways include a range of other primary and secondary metabolites in addition to lignin. Regulation of the various aspects of lignification will not only affect its downstream aspects but also may lead to the overproduction of other phenylpropane and glycoside derivatives, disrupting the original metabolic balance in plants and thus affecting normal plant growth and lignin accumulation.^32^ Therefore, the regulation of lignification by altering metabolic fluxes on relevant pathways needs to be examined from a systemic view. Based on a systematic understanding of plant methyl homeostasis and C-lignin polymerization mechanisms, searching for enzymes related to C-lignin synthesis with specificity is important breakthrough points to advance the regulation of C-lignin synthesis in the future.

## Isolation of C-lignin

Traditional lignin isolation methods include Klason analysis, acidic lithium bromide, enzymatic, organic solvent, and alkali treatment (Table 1).^15^ However, seeds usually contain a large amount of polysaccharides, acid-resistant lipids, and proteins. These compounds can be retained and mistakenly presented as lignin during the Klason method treatment.^12^ The alkali treatment method may also partially damage the structure of the benzodioxane linkage.^15^ Current pretreatment means for C-lignin in vanilla seeds and cactus seeds are enzymatic digestion coupling with mild acid treatment strategies (*e.g.*, cellulase coupling with acidic lithium bromide), which are related to the acid stability of benzodioxane bonds within C-lignin.^17^ After adequate crushing and acid pretreatment, C-lignin can be dissolved in organic solvents, which benefits subsequent processing and characterization.^17^ The seed coats of *Jatropha* are considered to be more economical and substantial sources of C-lignin than vanilla seeds. However, not only C-lignin but also G/S-type lignin are presented in *Jatropha*. Su and colleagues proposed a method for isolating C-lignin by simply crushing *Jatropha* seed bark and extracting C-lignin using dioxane with the help of dilute HCl.^12^ Dilute acid promotes the breaking of hydrogen bonds, thus preferentially releasing C-lignin with a low molecular weight into dioxane.^12^ However, the acidic environment will lead to the cleavage of the β-*O*-4 bond and thus dissolve the G/S lignin, which affects the product purity.^33^ Simple extraction using organic solvents suffers from the problem that only low-molecular-weight C-lignin can be extracted. Solvent polarity or the ability to interact with lignin (*e.g.*, to form hydrogen bonds) may be an important factor affecting the effectiveness of C-lignin extraction.^34,35^

**Table 1:** Comparison of different C-lignin isolation methods

Isolation methods	Materials	Pretreatment methods	Productivity	Advantages	Disadvantages	Ref.
Klason method	Vanilla seed bark	Ball mill + crude cellulase	>80%	Easy operation	Low product purity	11
Acid LiBr treatment	Vanilla seed bark	Pre-milled seed coats were treated with a modified Bligh and Dyer extraction method to remove oil and extracts	72.4%	Rapid removal of polysaccharides	Low product purity	17 and 38
Combined enzymatic and acid extraction	Castor seed bark	Enzymatic digestion	21%	Mild reaction conditions	Higher molecular weight and polydispersity index, low purity, high cost of enzymes	15
Alkali treatment	Castor seed bark	Particle size reduction	40%	Classic method, easy operation	Benzodioxane may be partially destroyed under alkaline conditions; irreversible condensation reactions exists	15
DMSO extraction	Vanilla seed bark	Ball mill + crude cellulase	24%	Rapid extraction, easy to operate	Only small molecular weight fractions are extracted	11
Dioxane/water extraction	*Jatropha* seed bark	Ball milling/simple grinding + crude cellulase	16%	Rapid extraction	Only small molecular weight fractions are extracted	11–13
Ethanol extraction	Castor seed bark	Ball mill + enzyme treatment	16.8%	Rapid extraction, easy to operate	Low productivity	15
DESs	Castor seed bark	Acetone/water extraction	41%	High purity, high recovery of solvents, high product selectivity	The interaction between DESs and C-lignin during the isolation process remains unclear	15

New green solvents that are flexible in changing properties (*e.g.*, viscosity, density, and polarity) hold great promise for efficient C-lignin extraction.^36^ Wang and coworkers applied deep eutectic solvents (DESs) to treat castor seed coats and found that the halogen anion Cl^−^ can form hydrogen bonds with –OH groups in lignin, resulting in lignin dissolution.^15,34^ DES acts as both a solvent and an acid catalyst for the cleavage of β-*O*-4 bonds. The molar concentration of caffeinated alcohol units in lignin obtained using DES was 1.87 μmol mg^−1^, corresponding to 31% of the isolated lignin by weight, which was higher than that with the acidic lithium bromide method.^15^ The designable anionic and side chain substituents of ionic liquids (ILs) make it promising to dissolve C-lignin by interacting with the benzene ring or hydroxyl group, which could facilitate C-lignin isolation.^35^ Although there are no examples of ILs applied to C-lignin separation, it should be possible to design ILs with stronger polarity and the ability to interact with C-lignin to develop new methods for C-lignin extraction. In addition, the co-solubilization of DES or ILs with other solvents to enhance the interaction between the solvents and C-lignin is an interesting approach.^37^ A more in-depth understanding of the interaction mechanisms would benefit the rational design of DESs and/or ILs solvents towards a more efficient C-lignin isolation.

## Catalytic depolymerization of C-lignin

**Fig. 3:**
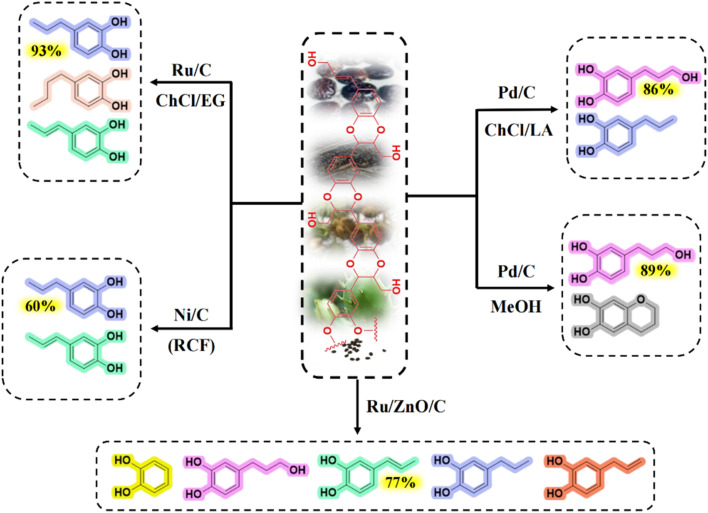
Product composition and major monomer product selectivity during catalysis of C-lignin with different metal catalysts.^16,24,43^

Thioglycolysis coupling RANEY® nickel desulfurization is one of the traditional methods for lignin depolymerization.^39,40^ The main advantages of thiolysis are the high selectivity for breaking reactions and easy identification of the degradation products.^17,41,42^ Alkaline nitrobenzene oxidation (NBO) is another traditional lignin depolymerization method.^17^ The principle of the NBO method is that the β-*O*-4 bond is stripped of formaldehyde under alkaline conditions to break ether bonds. Afterward, side-chain oxidation occurs to form aldehydes corresponding to the three lignin units, which can be used to identify trace differences in S/G units. Both thiolysis and alkaline oxidation require the participation of the free benzylic hydroxyl group on the lignin side chain. Since the benzylic group is not presented in C-lignin due to the stability of the benzodioxane structure, the conventional chemical degradation methods of lignin are ineffective for the depolymerization of C-lignin (monomer yield <1%).^17^ Metal catalysis hydrogenolysis is currently the dominant method of producing phenolic monomers from lignin. The benzodioxane bond in C-lignin can be completely cleaved by hydrogenolysis. The depolymerization products include aryl catechol, catechol propanol, catechol propane, *etc.*^12,16,17^ As shown in Fig. 3, the composition ratios of the monomer products using different catalysts and solvent combinations differed significantly. Li and coworkers showed that the side chain structure of the resulting monomers could be controlled by changing the catalysts and solvents.^17^ Metal catalyst-catalyzed hydrogenolysis has the advantages of high reactivity and selectivity, easy catalyst recovery, and can avoid catalyst deactivation during the hydrogenolysis reaction.^15,43^ The raw material for hydrogenolysis can be either the C-lignin extracted from the seed coats or the raw castor seed coats. The one-pot method with direct catalytic depolymerization of C-lignin-containing seed coats demonstrated a simplified reaction process over the two-step method, which achieved yield of 56.3 mg g^−1^ comparable to that of the two-step method.^44^

**Fig. 4:**
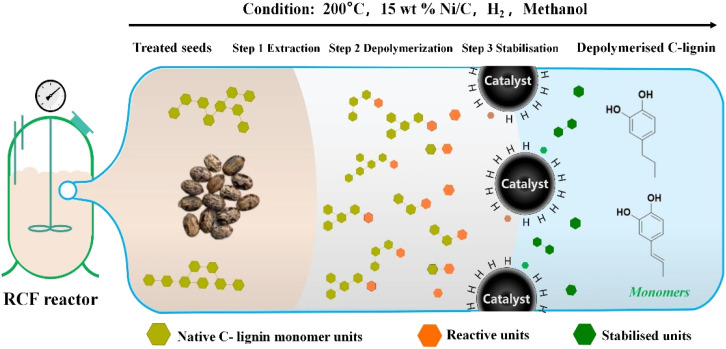
Reductive catalytic fractionation (RCF) process of C-lignin.^16^

When C-lignin was depolymerized by reductive catalytic fractionation (RCF), a polar hydrophilic solvent was used to extract lignin while the transition metal catalyst was used for reductive hydrogenolysis (Fig. 4). The ether bond was selectively cracked to improve the product uniformity. The depolymerization of C-lignin using the RCF method leads to a narrow distribution of monomer products (including only propyl and propenyl catechol). However, some issues, such as incomplete cleavage due to the catalyst inactivation, dilution of lignin oil by the extractant, and recondensation of lignin fragments during RCF, need to be addressed.^16^ Unlike general metal catalysts, atom-dispersed metals present higher catalytic activity and selectivity with more reusable times. Wang and colleagues used an atom-dispersed Ru catalyst (Zn-BTC metal–organic framework) to decompose the C–O bond in the benzodioxane bond, obtaining high selectivity (77%) for propenyl catechol production.^24^ Overall, depolymerization to produce catechol monomer is an important direction for the valorization of C-lignin. The efforts developing C-lignin depolymerization should be paid to improve the monomer yield and selectivity, reduce the economic cost, and optimize the downstream utilization.

## Valorization of C-lignin based on its homogeneous linear structure

### Depolymerization to produce fine chemical monomers

The primary method of industrial depolymerization of common lignin resources for producing phenolic monomers is thermochemical conversion, which requires severe conditions (*e.g.*, high temperature and high pressure), large energy consumption, and expensive facilities. Meanwhile, the product compositions are complex. For example, products of oxidative lignin cleavage include phenol, acetophenone, methyl benzoate, and benzoic acid.^45^ This result is mainly due to the heterogeneity and recalcitrant nature of common G/S lignin.^46^ The heterogeneity affects the selectivity of bond breakage and, thus, the monomer product compositions, which leads to difficulties in the subsequent product separation and purification. C-lignin's structure allows a simplified procedure for producing fine chemicals or other value-added products.^44^ The uniform benzodioxane structure benefits the higher monomeric phenol yields and selectivity during the depolymerization reactions. The high selectivity reduces the occurrence of unwanted side reactions and simplifies the subsequent purification.^24,47^ C-lignin presents more significant potential for producing homogeneous phenolic monomers than conventional G/S lignin.^48,49^

**Fig. 5:**
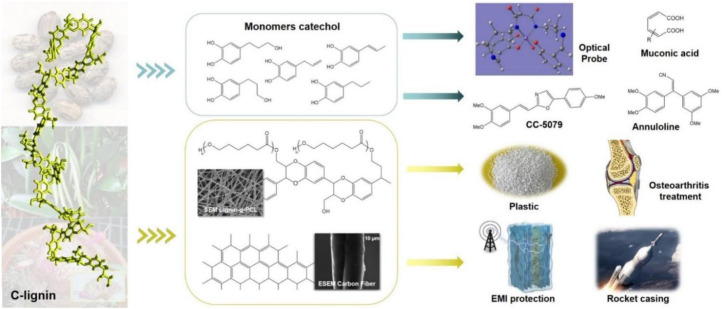
C-lignin application strategies and areas.^43,51–57^

Catechol monomers, which can be produced from C-lignin, are widely used in the chemical industry. Catechols and their derivative compounds are important precursors for the preparation of various functional composites (Fig. 5). End-chained catechols are important components of many bioactive molecules and drugs, as well as bionic functional materials.^24,50^ Various transformations of aryl catechol, the product of direct depolymerization of castor seed coat C-lignin, have been developed to obtain a series of functional molecular backbones involved in the current synthetic routes for the preparation of drugs and bioactive molecules. Song and coworkers used this C-lignin-derived compound from the castor seed coat, as a raw material for the facile and inexpensive synthesis of annuloline and CC-5079 (antitumor), demonstrating a promising application of C-lignin for drug synthesis.^51^ Catechol and its derivative compounds can also be used to detect Fe(iii) fluorescence.^52^ In addition, catechol can be cleaved by enzymatic ring-opening to generate mucofuranates, which are platform chemicals in industrial production.^24,53^ Overall, depolymerization of C-lignin through thermochemical reactions is rapid and efficient. However, the thermochemical reactions usually occurred under high temperature and high pressure, which require high energy and cost input. Biological depolymerization of C-lignin by enzymatic catalysis is environmentally friendly and the depolymerized products could be more homogenous.^54^ However, the production of the lignin-degrading enzymes is low at the current stage, which limits the large-scale application of biological depolymerization of C-lignin. By mimicking the enzymatic mechanisms, biomimetic catalysis is emerging through combining the advantages of both biological and chemical depolymerization methods, such as mild conditions and rapid reaction, which represents a promising approach for C-lignin depolymerization and conversion.

### Preparation of advanced polymer materials with C-lignin

Lignin has received attention as a precursor material for carbon fibers due to its high carbon content and relatively low cost. Lignin-based carbon fibers have a reasonably high specific surface area.^23^ C-lignin with high purity improves the performance of the corresponding carbon materials due to its excellent thermal stability and homogeneous structure.^15,24^ Nar and colleagues prepared carbon fibers by electrospinning C-lignin without additional modification or polymer blending.^33^ The resulting carbon fiber with a smaller diameter presented fewer impurities, higher thermal stability, and higher crystallinity than the typical carbon fibers made from kraft lignin.^33,43^ The unique benzodioxane bonding of C-lignin provided a more stable thermodynamic structure. Besides, the special benzodioxane bond allows C-lignin to exhibit unusual thermodynamic behavior when incorporated into polymeric materials. For example, grafting poly(ε-caprolactone) (PCL) on lignin hydroxyl groups allows the synthesis of lignin-*g*-PCL copolymers without adding any external solvents. Such copolymers have a wide range of promising applications in the plastics, composites, coatings, and pharmaceutical industries.^58^ The linear structure of C-lignin promotes the formation of different crystal morphologies in the copolymer. Given the unique linear aromatic structure of C-lignin and good thermal stability, the development of new polymeric materials is promising in the future.^58^

## Conclusion and outlook

**Fig. 6:**
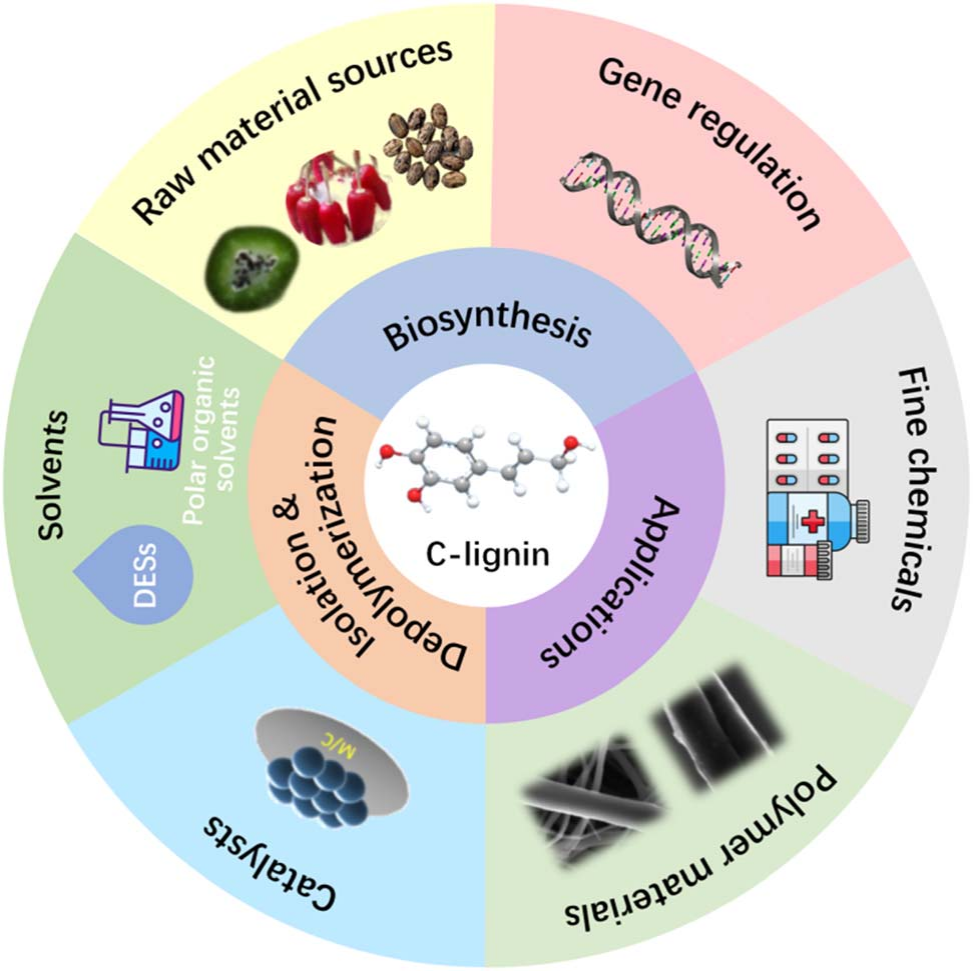
Summary of C-lignin biosynthesis, isolation, depolymerization, and application.

C-lignin is receiving increasing attention recently due to its homogeneous linear structure and narrow monomer product distribution through depolymerization. Expanding C-lignin feedstock sources and developing green and efficient extraction methods hold promise for promoting C-lignin valorization. The hope for reducing the cost of C-lignin feedstock is to regulate the biosynthesis of C-lignin in plants by genetic means (Fig. 6). Genetic engineering to regulate C-lignin synthesis requires systematic investigation of plant methyl homeostasis and caffeyl alcohol polymerization mechanism to address the issue that suppression of COMT and CcOAOMT genes affects plant growth and reduces total lignin contents. Traditional lignin isolation methods are not effective for C-lignin. Usually, hydrophilic polar solvents can be used to extract C-lignin from seed coats. DES treatment represents an effective method to isolate C-lignin, but the interaction between DES and C-lignin during the isolation process remains unclear. More efficient isolation methods need to be explored to provide C-lignin.

Currently, the utilization of C-lignin is mainly metal-catalyzed hydrolysis to produce catechol monomers and their derivatives. The narrow monomer product distribution makes C-lignin promising for producing fine chemicals. In addition, the good acid and thermal stability as well as the linear structure of C-lignin provides unique strength for the development of new polymeric materials, such as carbon fibers. The catechol compound is one of the key intermediates during the lignin bioconversion. The structure of depolymerized monomers from C-lignin is much like the catechol molecule, suggesting that C-lignin may be more suitable for biological valorization compared to the traditional G/S-type lignin. Therefore, the application of C-lignin in biotransformation holds promise for enhancing the biological lignin valorization performance. Exploration of a broader range of downstream applications is essential and promising for the future high-value utilization of C-lignin.

## Conflicts of interest

There are no conflicts to declare.

## Supplementary Material
